# The Geographical Conditioning of Regional Differentiation Characterising the COVID-19 Pandemic in European Countries

**DOI:** 10.3390/ijerph21101342

**Published:** 2024-10-10

**Authors:** Marcin Mazur, Jerzy Bański, Wioletta Kamińska

**Affiliations:** 1 Department of Rural Geography and Local Development, Institute of Geography and Spatial Organization, Polish Academy of Sciences, Twarda Str. 51/55, 00-818 Warsaw, Poland; jbanski@twarda.pan.pl; 2 Department of Socio-Economic Geography, Institute of Geography and Earth Sciences, Jan Kochanowski University of Kielce, Uniwersytecka Str. 7, 25-406 Kielce, Poland; wioletta.kaminska@ujk.edu.pl

**Keywords:** COVID-19 pandemic, European regions, geographical conditions, multiple regression

## Abstract

The aim of this paper is to assess the influence of selected geographical factors on the diversity of the development of the COVID-19 pandemic in Europe’s regions, and on its dynamics across the continent. The work took into account 250 of NUTS-2 regions. The datasets included the course of the COVID-19 pandemic (two dependent variables), intervening actions (four variables of the research background), and potential environmental and socio-economic conditioning (twelve independent variables). The dependent variables’ set was composed of two indexes: morbidity and temporal inertia. The temporal scope of the research was 23 March 2020–15 May 2022, with weekly resolution. By means of multiple linear regression model, the influence of the administrative actions and of the selected natural and socio-economic factors was assessed. Finally, a synthetic Regional Epidemic Vulnerability Index (REVI) for each individual region was calculated. It allowed us to classify the regions into three categories: resistant, neutral, or sensitive. REVI’s spatial distribution indicates that the zone of above-average vulnerability occurred in the western part of Europe and around the Alps. Therefore, focus ought to extend beyond regional statistics, towards spatial relationships, like contiguous or transit position. This research also validated the strong impact of national borders.

## 1. Introduction

December 2019 brought media reporting of a dangerous virus called SARS-CoV-2, causing the disease which was soon named COVID-19. The virus was first identified in the city of Wuhan (China), but then began its spread around the world, to the extent that the epidemic assumed the form of a true pandemic. Europe’s first known case of infection with the novel coronavirus was reported as early as on 25 January 2020, in France; and within the next few days, the problem was noted in other countries around the continent. Of the countries in the world experiencing more than a 100-fold increase in morbidity 15 days on from the identification of the first case, as many as 15 were in Europe [1]. Europe rapidly became an epicentre for the development of the disease.

The pandemic was soon subject to scientific research from every possible point of view, and geography was not left out of this process. Indeed, it has regularly served as a foundation for analytical work carried out by scientists in other fields. A compilation of the literature in the fields of geography, planning, and urban studies dating from autumn 2020—so less than a year into the outbreak of the disease—already revealed some 556 papers offering preliminary analysis of the COVID-19 pandemic [2]. There were also special editions of journals devoted to the analysis of its development, its consequences, and the methods used to study it [3,4]. Within the rapidly growing number of geographical studies on the development and consequences of the pandemic, three thrusts can be distinguished [5,6]. They relate to (i) the spatial diffusion of COVID-19, (ii) demography and (geo)political and economic implications of the pandemics, and (iii) forecasts of its impact on social and technological change, and territorial structure.

Thanks to this ever richer subject literature, as well as access to up-to-date and ever fuller and more accurate statistical materials, geographically conceptualised studies on the conditioning of the pandemic have become more and more detailed, and also diverse. Research has, for example, considered territorially differentiated symptoms of COVID-19 [7], or the levels of morbidity and mortality to be noted among different races, and ethnic and social groups in relation to geographical locations [8,9]. Analysis has also addressed regional differences in economic structure and commercial links as these affected the development of the pandemic [10], as well as factors linking up with different countries’ health policies, not least as regards actions seeking to stem pandemic development. Thus, work on differences in COVID-19 mortality from one region of Europe to another involved authors indicating how effective and decentralised management in health services as well as the rapid introduction of measures to limit the spread might prove to be very important factors holding down excess deaths [11].

From the very outset, a popular subject for research was territorial differentiation in features and effects of the pandemic, as analysed on the local, regional, and national scales [12,13,14,15,16,17,18]. This was accompanied by work on spatial relationships between the spread of COVID-19 and defined natural and socio-economic conditioning [19,20,21,22,23,24]. Where that natural conditioning is concerned, most coverage has been devoted to meteorological and climatic factors [25,26,27,28,29,30,31,32]. On the other hand, when it comes to socio-economic conditioning, a great deal of attention has been paid to demographics [8,33,34,35], mobility in society [36,37,38,39,40,41,42], and COVID-related policy pursued by states and regions [11,43,44]. The studies referred to above have been of considerable applied significance, as well as being important from a cognitive point of view. This is because they allow for the development of conclusions and recommendations for different units of territorial administration, where the constant aim is to curb the development and effects of COVID-19 (or an epidemic of some other disease that might well be encountered in future).

Among those seeking to ascertain the geographical variability of COVID-19 as a disease, widespread use has been made of diagnostic features assuming the form of indices potentially accounting for the territorial differentiation of both morbidity and mortality. One study of morbidity in Europe’s regions made use of a set of indices characterising the density of population, population age structure, longevity, GDP, technological equipping of hospitals, level of education, management system, and type of region [43]. Work on units of administration in Sweden made use of a broader range of potential factors that might account for COVID-19’s spatial differentiation. These included density of population, ages of inhabitants, shares of all inhabitants aged 70+ or 80+, sizes of household, connections by air, race and ethnic origin, level of unemployment, education, level of healthcare provisioning (including care homes), temperature, and the climate [34]. In research on COVID-related differences present among over 2500 Brazilian cities [45], the conclusions reached as regards the causes were different, even though quite similar variables had again been considered (i.e., population density, commuting to work, inhabitants over 60, care homes, household incomes, level of education, race of inhabitants, density of households in communities of the Favela type, and access to airports). Spatial research into the conditioning of the pandemic’s occurrence from one county to another across the USA was achieved by reference to differences in values for socio-economic variables (i.e., age, degree of disability, language, race, profession, and urban status), as set against data for numbers of cases and deaths in the first half of 2020 [46]. In turn, in India, interesting results were offered by work seeking relationships between the spread of the novel coronavirus and the means of organisation of the market put into effect in different districts [47]. The review in question makes it clear that factors behind the spread and effects of the pandemic are subject to clear geographical differentiation, to the point where conclusions to be drawn from analyses in different spatial units and scales can readily contradict one another. This may reflect the use of different measurement indices, or the quality of source material, or, of course, the methods applied in research [48]. A need thus arises for further study, with this needing to take particular account of comparative work involving large collections of units that can allow more objective results to be obtained.

The main objective of this article is to assess the influence of selected natural and socio-economic factors on regional differences in the development of the COVID-19 pandemic on the European continent. Its greatest novelty is the comparative approach and fairly widespread sample of the spatial units under the study, which allows for a more reliable assessment of the position of individual regions or groups of regions, as it provides a point of reference in the form of other regions. The authors are aware that the complex nature of the issue under study and the relatively short period of its analysis mean that the range of variables used here does not allow for the construction of a single universal and yet precise model. There are many conditions unique and specific to individual regions, as well as many soft factors difficult to quantify. On the other hand, however, such a model allows key relationships to be identified that, even if they do not sufficiently explain the spread of the pandemic, are so widely valid that their impact can be evidenced using quantitative methods.

In the following sections, the empirical material used is presented and the research design explained, followed by the presentation of the results, in quantitative and spatial terms. The former allows the overall model to be described and the relevance of individual conditions to be assessed, while the latter, using cartographic methods of presentation, makes it possible to identify patterns in the distribution of regions with similar or different vulnerability to pandemic spread.

The identification and assessment of the aforementioned determinants are then intended to enable the classification of EU regions into three key categories that differ in their vulnerability to pandemic development, namely, resilient, moderately resilient, and above-average vulnerable regions. Finally, the results obtained are critically evaluated and discussed.

## 2. Materials and Methods

In the further stages of the research procedure, 250 so-called NUTS-2 regions were taken into account. Out of all 257 such regions located in the territory of the EU and EFTA Member States in 2021, for 7 spatial units from Switzerland, the available data on the course of the pandemic did not meet the accepted standards. In addition, for some regions of Belgium, France, and Germany, as well as the Mazowieckie region in Poland, only data at a higher level of spatial aggregation, related to more than one NUTS-2 unit, were available. In their case, the pandemic data were spatially disaggregated between their subdivisions of NUTS-2 regions before proceeding further with the research procedure, using the following assumptions:

- The intensity of a phenomenon (e.g., numbers of cases per 1000 inhabitants) was uniform in each of a region’s NUTS-2 units);
- The absolute scale of a phenomenon (e.g., the total number of infections) might be subject to allocation in proportion with shares of a region’s population present in each NUTS-2 unit.

Prior to engagement in the research procedure, 3 datasets were compiled:

- On the course of the COVID-19 pandemic;
- On intervening actions that administrations took to curtail the spread of the disease in different regions and countries;
- On the potential geographical conditioning of the development of the pandemic.

The first dataset represents source material in line with which to calculate the indicators analysed as dependent variables. Data of the second group allow for the standardisation of the source material in line with different kinds of interventions, often emphasised as a key factor not linked directly to geographical conditions [49,50,51,52,53,54]. These are then data serving as intermediate variables (background and circumstances relating to the research). Data of the third group are a set of explanatory variables, offering an expression of factors identified by reference to the subject literature as representing potential conditioning linked with the geographical distribution and occurrence of foci of COVID-19.

The analysed database on the course of the COVID-19 pandemic in the regions is based on the data which originate from the national official sources (Table 1). They are collected and shared by the European Centre for Disease Prevention and Control (ECDC) at: https://www.ecdc.europa.eu/en/publications-data/sources-eueea-regional-data-covid-19 (accessed on 31 July 2024). These data allowed the period from 23 March 2020 (given that the start of the pandemic was announced by the WHO on 11th March) to mid-May 2022 to be covered. A weekly level of temporal resolution was chosen, with the study period covering 112 weeks, from week 13 of 2020 to week 19 of 2022. It was developed for the following two diagnostic indices:

- ${I1.1}_{i}=\sum_{w=1}^{112}{I1.1}_{i,w}$: the index of morbidity, whereby the total number of cases of COVID-19 infections w was noted in all weeks of research per 1000 inhabitants of a spatial unit i. The value for this index thus describes the overall intensity of COVID-19 in the given area.
- ${I1.2}_{i}=w_{max,i}-w_{max}$: the index of temporal inertia concerns the average number of days of temporal displacement of each of the three COVID-19 pandemic waves in a given spatial unit, as compared to the characteristics of these waves in the entire study area. This indicator aims to assess the dynamics of the pandemic phenomenon over time and therefore indicates the ability of individual regions to prepare for the arrival of the threat.

Therefore, the first dataset contains a determination of the index of morbidity (${I1.1}_{i,w}$) values in each of the 112 weeks (w), of one summary value for this (${I1.1}_{i}$), and of one value for the index of temporal inertia (${I1.2}_{i}$). Successive weeks (w) were marked with natural numbers in the range ”1” for the 13th week of 2020, through to ”112” for the last week of the temporal scope.

The second dataset is based on information on the various kinds of interventions undertaken in each particular region (i) and week (w), collected originally by the relevant national agencies (Table 1). It was provided again by the ECDC at: https://www.ecdc.europa.eu/en/publications-data/download-data-response-measures-covid-19 (accessed on 31 July 2024). However, it is possible that not all relevant administrative activities taken gained inclusion. This database allowed for the identification of 66 different kinds of actions. Often, the complex solutions were distinguished there as separate kinds of activity, running simultaneously through the same days. The 66 kinds mentioned are detailed enough to be classified, for the purpose of this study, exclusively and quite unambiguously into one of the four following categories:

- ${Int1}_{i,w}$: the closure of public venues;
- ${Int2}_{i,w}$: recommendations to stay at home;
- ${Int3}_{i,w}$: obligations to wear protective masks;
- ${Int4}_{i,w}$: online working or adaptation of workplaces.

In the case of composite actions, two or more of the four groups specified were then seen as active.

The third dataset comprises indicators that relate to potential geographical conditioning capable of differentiating the spread of COVID-19. These were selected by way of literature review and expert knowledge. The descriptions are as follows:

- ${I2.1}_{i}$ is the population density in 2021, according to Eurostat;
- ${I2.2}_{i}$ is the 2020 degree of urbanisation index, provided jointly by the Organisation for Economic Cooperation and Development (OECD), the European Commission’s Directorates General for Regional and Urban Policy (DG REGIO) and for Agriculture and Rural Development (DG AGRI), Eurostat, and the Joint Research Centre (JRC), abbreviated as DEGURBA, and based on the share of inhabitants living in “predominantly urban” or “intermediate” LAUs;
- ${I2.3}_{i}$ is the number of inhabitants within Functional Urban Areas (FUAs) delineated by Eurostat in 2019;
- ${I2.4}_{i}$ is the percentage of inhabitants aged 65 or above in 2021, according to Eurostat;
- ${I2.5}_{i}$ is the total number of interregional migrations for 2010–2018, per 1000 inhabitants, provided as a result of the Interregional Relations in Europe project (IRiE), financed by the European Observation Network for Territorial Development and Cohesion (ESPON);
- ${I2.6}_{i}$ is the percentage of inhabitants with higher education in 2021, according to Eurostat;
- ${I2.7}_{i}$ is GDP (PPP) per inhabitant in 2020, according to Eurostat;
- ${I2.8}_{i}$ is the type of region in the ESPON typology for mountainous regions, where:
  ⚬ 0 is assigned where more than half of the area is taken by regions classified as “non-mountain”;
  ⚬ 1 is assigned where the predominant area is taken by regions classified as ”mountain area”, even if inhabitants live mostly in others;
  ⚬ 2 is assigned where the predominant area is taken by regions classified as “mountain area”, and the predominant number of inhabitants live there.
- ${I2.9}_{i}$ is the mean annual air temperature for 2011–2020, according to Eurostat;
- ${I2.10}_{i}$ is the mean annual precipitation total for 2011–2020, according to Eurostat;
- ${I2.11}_{i}$ is the share of the area taken by forest in 2018, provided within Land Use/Cover Area frame statistical Survey (LUCAS) by Eurostat in close cooperation with the Directorate General responsible for Agriculture and the technical support of the JRC;
- ${I2.12}_{i}$ is the level of PM 2.5 air pollution in 2019, according to OECD.

The first three indices relate to features of a region’s settlement network. The next three concern demographic and societal aspects (age, mobility, and potential consciousness). The ${I2.7}_{i}$ index is then a reflection of economic opportunities, while the five remaining ones are reflections of diverse kinds of conditioning linking up with the state of the environment. Variables are diversified in terms of the cross-section or range over time, both due to constraints on data availability and the authors’ concern to limit the risk of being affected by the specificity of a particular year’s situation when a variable shows a significant variation in value from year to year and it is possible to calculate an average over a longer period. Each variable illustrates a different feature and carries different information in terms of substantive content. In controversial cases, merit incentives and the need to take into account all the most important features of the region that could significantly affect the course of the pandemic were given priority over technical standards of mutual co-linearity of independent variables [55,56,57]. Therefore, sometimes, in the absence of an alternative to a variable illustrating a certain feature that would meet the accepted standards of coverage and spatial resolution, a certain amount of multicollinearity was accepted (Table 2).

**Figure 1:**
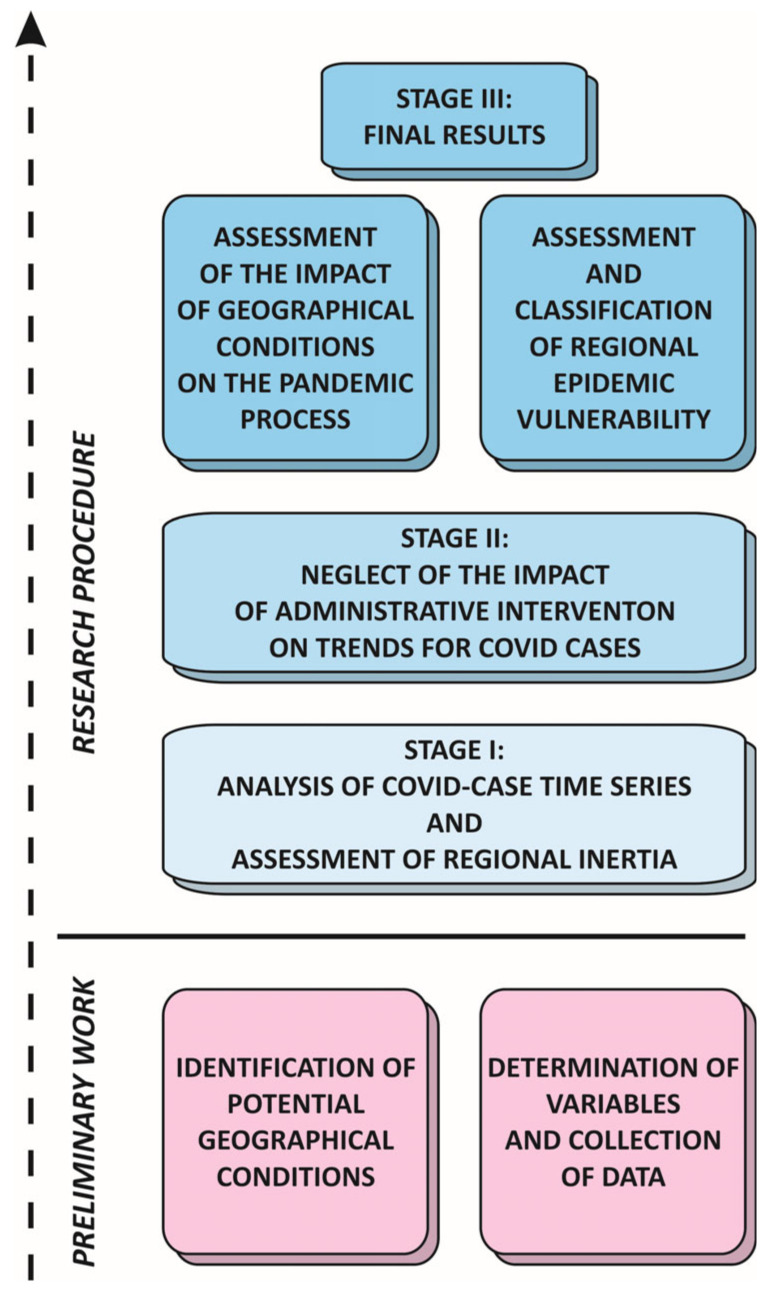
Stages of the research procedure.

The research procedure comprised three stages (Figure 1). In the first of these, a time-series analysis was carried out in regard to values of the index ${I1.1}_{i,w}$. This assured the achievement of two objectives. The first was the division of the entire temporal scope into separate identifiable sub-periods. Boundaries between three waves and their individual dates of culminations were identified. This provided the value to be assigned as the ${I1.2}_{i}$ index. The boundaries for the waves were indicated a priori, by reference to weeks in which local minimum numbers of cases within entire sets of units were recorded. Then, the reference dates of waves’ culminations for the study areas were determined, as well as individual dates for each individual region. This was defined as the half-way point for the given wave’s total number of infections. The final index value was constituted by the mean difference between individual and reference date of culmination for three waves.

The second objective was to cross-validate the empirical dynamics of weekly number of cases, in order to determine by means of LSM the predictive model, which considers the dynamics of infection numbers in a given spatial unit in a certain number of following weeks at given dynamics noted in the previous week. For the calculation of dynamics in the week w, the following formula was applied:

(1) $${I1.1}_{i,w}Din=\frac{{I1.1}_{i,w}}{{I1.1}_{i,w-1}}-1$$

A consecutive increase in the number of forecasted weeks usually implies an increase in fit obtained, although at the expense of decrease in cases available to be analysed. In this trade-off, the optimal, in terms of *p*-value, number of following weeks to be taken into consideration was established. The minimal value of $p=1.368\times 10^{-30}$ was obtained when the three following weeks were forecasted (r=0.073 while 24,984 forecasts were taken into account). This model was used in the second stage of the research procedure, to estimate and omit the impact of particular categories of actions taken by the public administrations on the numbers of infections reported, by taking into consideration these four additional explanatory variables. It was assumed that the empirical evidence of a relatively smaller number of infections following administrative intervention taken in previous weeks would indicate its positive effect.

The second stage of the research procedure entailed the possible discounting of the actions taken by the administrations, when it came to numbers of cases of COVID-19. In this stage, a predictive multiple linear regression model was developed by involving various scenarios of interventions being applied in previous weeks. It would allow to discount their impact and to focus on the impact of environmental and socio-economic conditions in the last stage.

The third stage of the research procedure ensured the achievement of two further objectives. The first was to assess the influence of selected environmental and socio-economic factors on interregional differences in both the level of development and the dynamics of the experienced pandemic. The regression model describing the pandemic’s trajectory in relation to a set of indices depicting potential geographical conditioning in the spatial units was used to indicate the factors which played a significant role. The remaining objective was pursued by constructing an original synthetic Regional Epidemic Vulnerability Index (REVI) and calculating its values for each individual region i. This drew on both the probability of infection and time to prepare for the strike of the pandemic across European regions. The calculation used the formula:

(2) $${REVI}_{i}=\frac{\frac{{I1.1}_{i}-\bar{I1.1}}{\sigma_{I1.1}}-\frac{{I1.2}_{i}-\bar{I1.2}}{\sigma_{I1.2}}}{2}$$

In formal terms, the synthetic indicator is the arithmetic mean of the standardised value of the deviation from the mean of its two components, i.e., the index of morbidity and the index of temporal inertia, taking into account that one component is a stimulant and the other a destimulant. As a result of the standardisation, in both cases, the value of a sub-index in a given region is related not only to the values in the other regions forming the analysed set, but also to the context of that index’s variability. In this way, an equal impact of both components on the final value of the synthetic indicator is obtained. Its reference level is zero, indicating that the state of one component is as favourable against the background of other regions as the state of another is unfavourable. The value for this index was not normalised nor confined by some maximum or minimum assumed in advance.

## 3. Results

**Figure 2:**
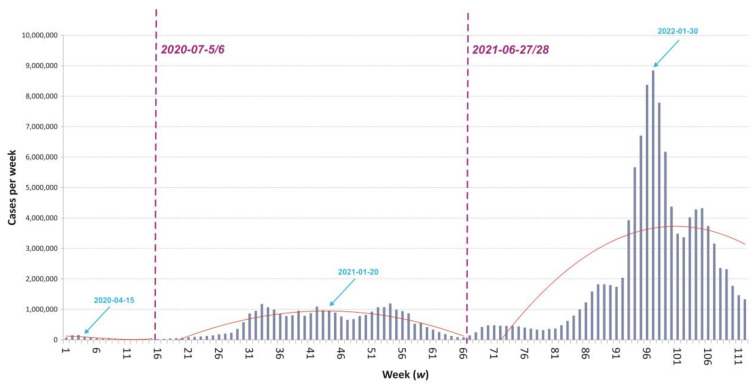
The periods of the pandemic course in Europe against the background of weekly numbers of COVID-19 infections. Limit dates between waves are indicated in red, along with (in blue) the determined dates on which half of all cases in that wave were recorded. Source: authors’ own elaboration based on data from the European Centre for Disease Prevention and Control.

The entire temporal scope under analysis has been divided into periods, in line with the phases of the pandemic identified (Figure 2). Technical trend analysis employed in, e.g., market trend studies [58] was employed for periodisation. As the potential boundaries between successive waves may be indicated by the local minima of the weekly number of new infections in the entire study area, i.e., values that are lower than both the number found in the previous week and in the following week, the trend analysis was limited to a sequence based on these minima, the so-called support level. The monotonicity of the sequence of all 11 local minima found changes three times, from decreasing to increasing, again to decreasing, and again to increasing. On the basis of the two changes in monotonicity from decreasing to increasing, it can be assumed that three waves of infection were registered in the entire study area during the observation period. The two local minima at which this change in monotonicity occurred, week 15 and week 66, with 21.5th. and 84.7th. new cases of infections, respectively, represent their limits. The reference dates for the consecutive phases of the pandemic are respectively 15 April 2020, 20 January 2021, and 30 January 2022.

The temporal breakdown of the data indicates a clear regularity that successive waves were associated with ever greater numbers of people infected, and, in consequence, with greater numbers of deaths due to COVID-19. This trend is to be observed in the entire set of 250 regions (Table 3), as well as in every individual country analysed. At the same time, the risk of health consequences actually declined with each new wave. Thus, the second wave brought 28 times as many reported infections in the study area as the first wave, even as numbers of deaths were just under 5 times as high. This decline in numbers of deaths as related to numbers of reported cases was present in almost all 30 countries, with the exception of Slovakia only. Furthermore, while almost 3.5 times as many infections were evidenced during the third wave as during the second one, even the absolute number of deaths due to COVID-19 was significantly lowered. The fall in the numbers of deaths per 1000 people infected was noticeable in every country.

**Figure 3:**
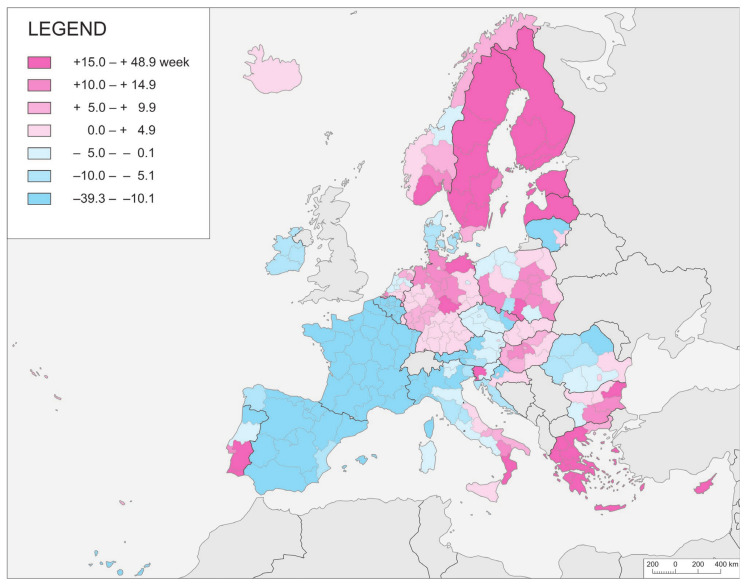
NUTS-2 regions by index of temporal inertia (${I1.2}_{i}$). Given the lack of data from all of France’s regions, the shift from Wave I of the pandemic to Wave II was taken as given by a value calculated by reference to numbers of cases in the country as a whole.

NUTS-2 regions in different countries are often markedly differentiated in terms of the temporal inertia index, even though they are territorially contiguous (Figure 3). It was in the cases of Sweden, Finland, Estonia, Latvia, and Greece that the waves of the pandemic made their latest appearances, while in Spain, France, Ireland, Lithuania, and Hungary they arrived sooner. Intra-national differences are to be noted in the remaining countries. This rather fuzzy spatial regularity, accompanied, in general, by a quite clear border effect, seems to be meaningful. This may reflect the diverse domestic methodologies used to gather statistical materials, or else be an effect of nationwide intervention measures taken in all regions at the same time.

After the accommodation of a further four variables related to categories of administrative intervention (${Int1}_{i,w}$ … ${Int4}_{i,w}$), the model estimated the number of cases slightly more significantly, but it did not substantially improve the estimation, as the r-value only increased from 0.073 to 0.080 (Table 4). It is meaningful that the statistical impact of online working or adaptation of workplaces (${Int4}_{i,w}$) is even negative, and the impact of obligations to wear protective masks (${Int3}_{i,w}$) turned out to be insignificant and also could not support the consideration of administrative actions in the final model.

**Figure 4:**
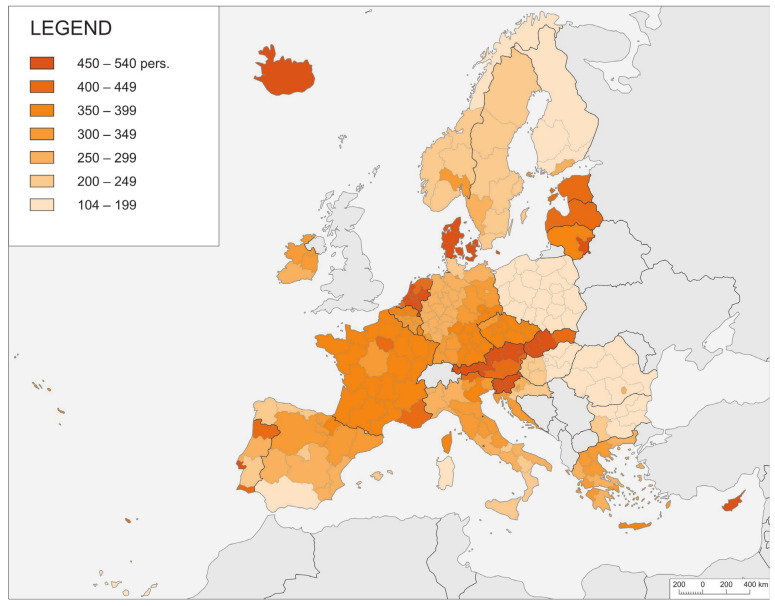
NUTS-2 regions by index of morbidity (${I1.1}_{i}$).

Morbidity shows significant spatial diversity, with the highest values for the index being noted during the third wave of the pandemic (Figure 4). In Cyprus and Iceland, as well as Denmark and Austria, the index assumed a total value of more than 400 cases per 1000 inhabitants. This means that more than two infections were noted during the period of research among every five citizens. On the other hand, in Finland, Poland, eastern Hungary, Romania, and Bulgaria, the total value never exceeded 200. In general, it can be noted how this value is lower in Eastern Europe, and, to some extent, also in Scandinavia or the southernmost regions. An exception was provided by the Baltic countries of Lithuania, Latvia, and Estonia, where rates were similar to those in either Central or Western Europe.

When it came to models describing the spatial variability of the two indices for the course of the COVID-19 pandemic in relation to geographical conditioning, the value of the coefficient of determination was not very high, regardless of whether the phases of the pandemic were studied separately or together (Table 5). Treated holistically, as a certain kind of system of geographical conditioning, the whole set of independent variables allows for some 29.4% of the variation in the index of morbidity to be explained, as well as 41.2% of the variation in the inertia index. In both cases, statistical significance was evidenced, thanks to the dataset size being large enough. Geographical conditioning determines more strongly the time of arrival of waves of the pandemic than it does the commonness of infection. In the case of the model explaining the spatial diversity of values for the morbidity index, it is possible to note a marked rise in goodness-of-fit along with passing to each successive wave. On the one hand, ever greater shares of the whole of society were encompassed during consecutive waves; on the other, these were more and more dependent on geographical conditioning. Moreover, temporal inertia also co-occurred most markedly with geographical conditioning during the most recent phase of the pandemic.

While the influence of the whole system of geographical conditioning was confirmed, any consideration of individual explanatory variables fails to give rise to such unequivocal conclusions (Table 6). Where the whole analysed period is taken into account, a higher number of infections and earlier development of the pandemic both co-occur with a more warm and humid type of climate, as well as a lower share of forest in land cover. Also, a lower degree of air pollution is significantly correlated with the faster and more extensive development of the pandemic, which might be surprising and is worth of deeper investigation. The other environmental conditions included in the analysis do not show a clear statistical relationship with measures of the course of the pandemic.

An interesting observation is that it is far rarer to observe a significant co-occurrence of indexes reflecting the course of the pandemic with socio-economic variables. A higher value of morbidity index is noted in richer regions and those with a higher share of tertiary-educated population. Yet the COVID-19 pandemic developed sooner in densely populated regions, those with smaller shares of elderly people, and those in which the intensity of migratory movements is lower.

**Figure 5:**
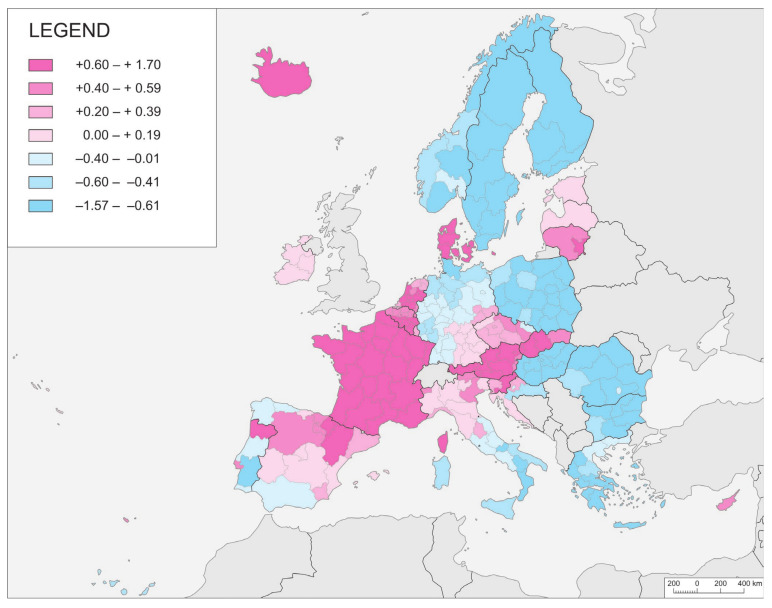
NUTS-2 regions and their values for the Regional Epidemic Vulnerability Index (${REVI}_{i}$).

A synthesis of the knowledge, gathered by providing the Regional Epidemic Vulnerability Index (${REVI}_{i}$) values (Figure 5), allowed the authors to note large continuous zones of above-average vulnerability to the spread of the pandemic. One such zone includes the western part of the study area, as well as an area extending across the centre of the continent around the Alps. In opposition to Western and Central Europe, the whole northern and eastern parts of the study area, as well as Europe’s far south, seem to be characterised by a relatively low level of vulnerability. The most prone areas are clearly identified as those with the largest exposure to inter- or trans-continental flows of people. One can also clearly notice a certain tendency to neighbour similarity and the negative correlation of this similarity with physical distance between regions (the so-called spatial autocorrelation). The only doubt raised here concerns the sharp difference in the ${REVI}_{i}$ value across state borders.

## 4. Discussion

The assessment of the studied natural and socio-economic factors’ impact on regional diversity in the development of the COVID-19 pandemic in Europe needs to be very comprehensive, so significant limitations at this point are inevitable. For instance, an important obstacle of the study is an incidental occurrence of the phenomenon. It occurs so rarely, thus in the context of so specific state of knowledge and readiness of the global community that it can even be considered unique. Important difficulties are also caused by the selection of essential independent variables that cover the explanatory content but, at the same time, do not duplicate each other’s contributions. This is, for instance, the case of the lack of information on the impact of diversified acquired immunity across regions caused by previous contact with virus. Yet, the coverage and resolution standards ought to be met to fulfil the assumptions of a comparative approach, both spatially and temporally. These obstacles are clearly proved by the results and the seemingly insufficient accuracy of the model. Many conditions still exist that stay beyond the model, either due to an inexhaustive list of potential geographical factors reflected in the set of independent variables, or caused by the specific nature of each individual region, which is impossible to capture entirely in one universal model. Yet, there also soft factors difficult to quantify. All of these conditions, whether omitted, of a regional nature, or difficult to capture quantitatively, are the potential directions for further in-depth research.

Nevertheless, the main objectives of this article can be considered to have been met. Through its novel comparability of results across regions throughout the continent, the model allowed for the detection of some general spatial regularities. Some of these should attract particular attention. It seems meaningful that the spatial regularity identified is clearly influenced by the pattern of country borders. This may be the effect of nationwide interventions undertaken in all regions of the country at the same time, the effects of which are emphasised by many authors [49,50,51,52,53,54], but may also reflect different methodologies of collecting statistical data in individual countries. In turn, the reasons for the lack of significant improvement in the model parameters resulting from the inclusion of different administrative interventions can be found in the existence of feedback [50]. These interventions co-occur with an increase in the number of infections detected, as they are decided upon in response to this rise. Any positive impact they undoubtedly have only becomes apparent on a statistically observable scale after some time. However, given the complexity of the system of multiple interacting determinants, it is difficult to isolate the scale of their net effect or the period of impact. A deeper insight into these ambiguous results goes beyond the purpose of this study, but provides an important rationale for further research directions on comparing regional vulnerability to the pandemic spread.

It is noteworthy, moreover, that the indicators of the course of a pandemic, and, in particular, the temporal inertia of its spread increasingly co-occur with geographical determinants in each successive phase of the pandemic. The role of geographical determinants therefore increases as the commonness of infection increases. Even during the third wave of the pandemic, however, there are significant differences in the levels of incidence in neighbouring countries that are difficult to explain unequivocally. How, for example, did such a difference emerge between Poland and its surrounding countries Lithuania, Slovakia, and Czechia (Figure 4 and Figure 5)? The countries mentioned do not differ much in terms of social or economic conditions. The reason for the differences can be found in the size of the countries, as, in general, the highest relative morbidity was rather observed in small countries. Even within this general pattern, there is a large and notable exception, namely, France, where the value of the indicator is well above average.

The dependencies shown for socio-economic conditions may be a reflection of the state of health services, as a higher number of COVID tests performed provides a greater chance of detecting viral infection [59,60]. However, the impact of the level of health service development on the number of infections may not only consist of more rigorous statistics, but may also be causal in nature. Previous studies have shown that during the first period of the pandemic, the healthcare sector was a significant promoter of infection [1]. The virus had very good transmission conditions and also encountered immunocompromised people in care homes [34]. Another explanation could also be that in richer and better educated societies, the population shows greater mobility [38], which is one of the key determinants of the pandemic spread [37].

Also the cause–effect relationship between air pollution and the COVID-19 pandemic is relatively often addressed in the literature [61,62,63,64]. Therefore, it is worth paying particular attention to a very interesting result obtained for the last of the geographical factors analysed (${I.12}_{i}$). It was shown that among European regions with higher PM 2.5 air pollution, significantly fewer infections in relation to the population were recorded, and the maximum level of subsequent pandemic waves occurred significantly earlier. However, it should be borne in mind that the model takes into account the influence of all 12 independent variables collectively, so it also takes into account a number of relationships between them. Thus, the fact that the smallest I1.1 value is to be noted in the CEECs, where the level of air pollution is relatively high, may reflect different features serving to distinguish this specific area from other parts of Europe. It can be determined by, for instance, a lower level of diagnosis of infections, the lower mobility of society, or other specific characteristics, which favour fewer observed cases of infection, although they co-occur in the area with high air pollution.

The characteristics of the spatial distribution of the synthetic Regional Epidemic Vulnerability Index (REVI) values, including, in particular, its entropy and spatial autocorrelation within countries, point to the relevance of research into general spatial regularities and, going beyond an analysis of the individual characteristics of each region, its specific socio-economic situation or the activities undertaken there. Future research should place more emphasis on the geographical determinants of the pandemic spread, including, in particular, the spatial relationships between the units under study, their physical proximity to each other, and the various natural (water bodies, mountain ranges, etc.) and socio-cultural (boundary permeability, language or currency differences, historical links, etc.) factors stimulating or limiting the interregional movement of people and thus potential virus transmission.

There are also several secondary findings from the study, which are of a general, non-spatial nature, but relevant to characterising the course of the pandemic in Europe. First of all, the observed overall mortality rate due to COVID-19 in the study area resembles that caused by seasonal influenza, i.e., a level in the range of 1–5% (https://www.gov.pl/web/gis/grypa-sezonowa) (accessed on 31 July 2024). Moreover, the mortality rate in each successive wave was clearly decreasing, despite the increase in the total number of deaths due to COVID-19 resulting from the increase in the absolute number of infections [32,65,66]. This may be explained by the proportion of the population vaccinated [59,65,67,68,69,70,71,72,73,74] and/or having acquired immunity as a result of infection [68,71], but also by the mutation of the virus towards variants less harmful to human health and life [65]. Among the potential reasons for the decrease in the rate of fatal COVID-19 infections, the statistical effect should not be overlooked, as the increasing use of tests [59,60] increases detection rates and allows statistics to reflect the actual situation more accurately [50,60].

## 5. Conclusions

The course of the COVID-19 pandemic, at least in Europe, is probably progressing towards the more widespread but less dangerous seasonal waves of disease known for years. The pandemic’s end point is therefore undoubtedly fuzzy and conventional, but in the light of the results obtained, it may be argued that a more useful approach is to use a certain conventional threshold in terms of the mortality caused by a particular type of virus rather than one expressed in incidence level. Such a proposal is even more justified when we note that there is probably more complete and precise recording of mortality than of morbidity.

The co-occurrence of the selected elements of natural environment with measures of the pandemic course proved that environmental conditions create a very complex system. Whereas a multiple regression model allows for the assessment of the significance of each explanatory variable if the status quo among the others is assumed, which might be very useful in terms of analytical purposes, this system still contains numerous mutual loops and feedbacks. It has thus to be accepted that each individual natural factor cannot be invoked individually to explain spatial differentiation in the pandemic course, but it rather ought to be perceived as an element of the system, which inherently interacts with others. Therefore, its extracted specific effect can be entirely inversed by its indirect impact as feedback which is given to the other elements of the system.

In general, a given wave of COVID-19 made its appearance earliest in the western part of the continent, while it was in northern and southeastern parts that the latest onsets were noted. However, where Central Europe was concerned, the situation was disparate, with variation noted from region to region. A more worthwhile conclusion might be drawn in relation to domestic differences characterising the index of temporal inertia. In some countries, all regions received successive waves of the pandemic almost at the same time, whereas, in others, it was possible to note distinct regional differences. This noticeably continuous spatial pattern of temporal inertia determines to a large extent the spatial distribution of the synthetic Regional Epidemic Vulnerability Index. It seems to indicate the special position of some kind of hub for flows of people. This provides an incentive to develop further research on the drivers of pandemic development at the interregional scale. Indeed, it is worth putting more emphasis on issues relating to the location of the region, the route of major transport routes, and the mobility of people.

## Figures and Tables

**Table 1:** National sources of data on the course of the COVID-19 pandemic.

Country	Source
Austria	https://www.sozialministerium.at/ (accessed on 31 July 2024)
Belgium	https://epistat.wiv-isp.be/covid/covid-19.html (accessed on 6 September 2022)
Bulgaria	https://covid19.lanix.org/ (accessed on 31 July 2024)
Croatia	https://www.ecdc.europa.eu/en/publications-data/european-surveillance-system-tessy (accessed on 6 September 2022)
Cyprus	https://www.ecdc.europa.eu/en/publications-data/european-surveillance-system-tessy (accessed on 31 July 2024)
Czechia	https://onemocneni-aktualne.mzcr.cz/covid-19 (accessed on 31 July 2024)
Denmark	https://www.ssi.dk/sygdomme-beredskab-og-forskning/sygdomsovervaagning/c/covid19-overvaagning (accessed on 31 July 2024)
Estonia	https://www.terviseamet.ee/et/koroonaviirus/koroonakaart (accessed on 31 July 2024)
Finland	https://experience.arcgis.com/experience/d40b2aaf08be4b9c8ec38de30b714f26 (accessed on 31 July 2024)
Germany	https://experience.arcgis.com/experience/478220a4c454480e823b17327b2bf1d4 (accessed on 31 July 2024)
Greece	https://covid19.gov.gr/covid19-live-analytics/ (accessed on 31 July 2024)
Hungary	https://www.ecdc.europa.eu/en/publications-data/european-surveillance-system-tessy (accessed on 31 July 2024)
Iceland	https://www.ecdc.europa.eu/en/publications-data/european-surveillance-system-tessy (accessed on 31 July 2024)
Ireland	https://www.ecdc.europa.eu/en/publications-data/european-surveillance-system-tessy (accessed on 31 July 2024)
Italy	https://www.arcgis.com/apps/opsdashboard/index.html#/b0c68bce2cce478eaac82fe38d4138b1 (accessed on 31 July 2024)
Latvia	https://data.gov.lv/dati/lv/dataset/covid-19-pa-adm-terit/resource/492931dd-0012-46d7-b415-76fe0ec7c216 (accessed on 31 July 2024)
Liechtenstein	https://www.ecdc.europa.eu/en/publications-data/european-surveillance-system-tessy (accessed on 31 July 2024)
Luxembourg	https://gouvernement.lu/fr/dossiers.gouv_msan+fr+dossiers+2020+corona-virus.html (accessed on 31 July 2024)
Malta	https://deputyprimeminister.gov.mt/en/health-promotion/Pages/Novel-coronavirus.aspx (accessed on 31 July 2024)
Netherlands	https://data.rivm.nl/covid-19/ (accessed on 31 July 2024)
Norway	https://www.fhi.no/en/id/infectious-diseases/coronavirus/daily-reports/daily-reports-COVID19/ (accessed on 31 July 2024)
Poland	https://www.gov.pl/web/koronawirus/wykaz-zarazen-koronawirusem-sars-cov-2 (accessed on 31 July 2024)
Portugal	https://covid19.min-saude.pt/ (accessed on 31 July 2024)
Romania	https://instnsp.maps.arcgis.com/apps/opsdashboard/index.html#/5eced796595b4ee585bcdba03e30c127 (accessed on 31 July 2024)
Slovakia	https://korona.gov.sk/koronavirus-na-slovensku-v-cislach/ (accessed on 31 July 2024)
Slovenia	https://www.nijz.si/sl/dnevno-spremljanje-okuzb-s-sars-cov-2-covid-19 (accessed on 31 July 2024)
Spain	https://www.mscbs.gob.es/profesionales/saludPublica/ccayes/alertasActual/nCov-China/situacionActual.htm (accessed on 31 July 2024)
Sweden	https://experience.arcgis.com/experience/2dc63e26f509468f896ec69476b0dab3 (accessed on 31 July 2024)

Source: European Centre for Disease Prevention and Control.

**Table 2:** Pearson’s coefficient of linear correlation across independent variables.

Variable	I2.1	I2.2	I2.3	I2.4	I2.5	I2.6	I2.7	I2.8	I2.9	I2.10	I2.11	I2.12
I2.1	1.000	0.451	0.163	−0.319	0.289	0.234	0.308	−0.154	0.128	−0.138	−0.307	0.254
I2.2	0.451	1.000	0.437	−0.271	0.117	0.269	0.350	−0.119	0.187	−0.109	−0.190	0.069
I2.3	0.163	0.437	1.000	−0.077	−0.059	0.240	0.299	−0.161	0.030	−0.060	−0.139	0.137
I2.4	−0.319	−0.271	−0.077	1.000	−0.061	−0.188	−0.177	0.057	−0.126	0.135	0.240	−0.132
I2.5	0.289	0.117	−0.059	−0.061	1.000	0.172	0.371	−0.214	−0.206	−0.094	−0.191	−0.121
I2.6	0.234	0.269	0.240	−0.188	0.172	1.000	0.600	−0.218	−0.259	−0.033	−0.120	−0.229
I2.7	0.308	0.350	0.299	−0.177	0.371	0.600	1.000	−0.188	−0.371	0.183	−0.315	−0.228
I2.8	−0.154	−0.119	−0.161	0.057	−0.214	−0.218	−0.188	1.000	0.063	0.438	0.481	−0.120
I2.9	0.128	0.187	0.030	−0.126	−0.206	−0.259	−0.371	0.063	1.000	−0.372	0.042	0.094
I2.10	−0.138	−0.109	−0.060	0.135	−0.094	−0.033	0.183	0.438	−0.372	1.000	0.177	−0.061
I2.11	−0.307	−0.190	−0.139	0.240	−0.191	−0.120	−0.315	0.481	0.042	0.177	1.000	−0.149
I2.12	0.254	0.069	0.137	−0.132	−0.121	−0.229	−0.228	−0.120	0.094	−0.061	−0.149	1.000

Source: authors’ own elaboration.

**Table 3:** Numbers of infections and deaths caused by COVID-19 in the study area, according to waves of the pandemic.

Infected people					
Number			Per 1000 inhabitants		
Wave I	Wave II	Wave III	Wave I	Wave II	Wave III
1,107,017	31,365,731	107,841,356	2.4	69.2	238.1
Deaths					
Number			Per 1000 infected		
Wave I	Wave II	Wave III	Wave I	Wave II	Wave III
127,415	608,822	345,899	115.1	19.4	3.2

Source: authors’ own elaboration based on data from the European Centre for Disease Prevention and Control.

**Table 4:** Statistics of the predictive model, after including four categories of administrative intervention as independent variables: individual coefficients (b) of variables, their limit levels of statistical significance (*p*-value), coefficient of correlation (r), set size (n), and *p*-value of the entire model. Coefficients describing situations in which statistical significance was achieved are as highlighted.

Independent Variables				
Statistic measure	b		*p*-value	
Dynamics of 1st following week	0.037		<10^−30^	
Dynamics of 2nd following week	0.046		<10^−30^	
Dynamics of 3rd following week	0.024		1.880 × 10^−4^	
${Int1}_{i,w}$	6.930		4.682 × 10^−2^	
${Int2}_{i,w}$	20.908		4.000 × 10^−6^	
${Int3}_{i,w}$	−1.644		5.123 × 10^−1^	
${Int4}_{i,w}$	−6.784		7.010 × 10^−4^	
Constant term	−5.953		1.824 × 10^−1^	
Model				
Statistic measure	R^2^	r	n	*p*-value
Value	0.006	0.080	25.259	1.423 × 10^−31^

Source: authors’ own elaboration.

**Table 5:** Values of the $R^{2}$ coefficients of determination for both variables explained.

	I1.1				I1.2			
	Total	Wave I	Wave II	Wave III	Total	Wave I	Wave II	Wave III
$R^{2}$	0.294	0.213	0.318	0.381	0.412	0.320	0.312	0.406
r	0.542	0.462	0.564	0.617	0.642	0.566	0.559	0.637
n	228	228	228	228	228	228	228	228
*p*-value	2.485 × 10^−20^	4.185 × 10^−14^	1.339 × 10^−21^	1.598 × 10^−26^	2.558 × 10^−23^	3.370 × 10^−21^	3.614 × 10^−18^	1.838 × 10^−27^

Source: authors’ own elaboration.

**Table 6:** Coefficients for the regression functions in models describing the indices of morbidity and temporal inertia (b), as well as their limit levels of statistical significance (*p*-value). Coefficients describing situations in which statistical significance was achieved are as highlighted.

	b		*p*-Value	
	I1.1	I1.2	I1.1	I1.2
I2.1	−0.001	−0.003	0.943	0.035
I2.2	−44.668	7.838	0.291	0.238
I2.3	0.000	0.000	0.166	0.095
I2.4	263.337	67.312	0.170	0.026
I2.5	−0.007	0.003	0.051	0.000
I2.6	184.013	−20.902	0.015	0.077
I2.7	0.003	0.000	0.001	0.230
I2.8	21.162	1.258	0.044	0.444
I2.9	5.896	−1.000	0.002	0.001
I2.10	0.063	−0.025	0.033	0.000
I2.11	−164.454	40.236	0.000	0.000
I2.12	−3.376	0.863	0.022	0.000
Constant term	156.203	−22.217	0.943	0.035

Source: authors’ own elaboration.

## Data Availability

All data listed or described in the Materials and Methods section are free and publicly accessible.
